# Repurposing *Castanea sativa* Spiny Burr By-Products Extract as a Potentially Effective Anti-Inflammatory Agent for Novel Future Biotechnological Applications

**DOI:** 10.3390/life14060763

**Published:** 2024-06-15

**Authors:** Luisa Frusciante, Michela Geminiani, Tommaso Olmastroni, Pierfrancesco Mastroeni, Alfonso Trezza, Laura Salvini, Stefania Lamponi, Ottavia Spiga, Annalisa Santucci

**Affiliations:** 1 Dipartimento di Biotecnologie Chimica e Farmacia, Università di Siena, Via Aldo Moro, 53100 Siena, Italy; luisa.frusciante@unisi.it (L.F.); tommaso.olmastroni@student.unisi.it (T.O.); p.mastroeni@student.unisi.it (P.M.); alfonso.trezza2@unisi.it (A.T.); stefania.lamponi@unisi.it (S.L.); ottavia.spiga@unisi.it (O.S.); annalisa.santucci@unisi.it (A.S.); 2 SienabioACTIVE, Università di Siena, Via Aldo Moro, 53100 Siena, Italy; 3 Fondazione Toscana Life Sciences, Strada del Petriccio e Belriguardo, 53100 Siena, Italy; l.salvini@toscanalifesciences.org; 4 ARTES 4.0, Viale Rinaldo Piaggio, 34, 56025 Pontedera, Italy

**Keywords:** *Castanea sativa*, waste repurposing, polyphenols, UPLC-MS/MS, inflammation, RAW 264.7, molecular modeling, docking simulations, spiny burrs

## Abstract

The concept of a “circular bioeconomy” holds great promise for the health, cosmetic, and nutrition sectors by re-using *Castanea sativa* (Mill.) by-products. This sustainable resource is rich in bioactive secondary metabolites with antioxidant and anti-inflammatory properties. By transforming these by-products into high-value products for human health, we can promote sustainable economic growth and reduce the environmental impact of traditional waste disposal, adding value to previously underutilized resources. In the present study, we investigated the antioxidant capacity, phytochemical composition, and in vitro antioxidant and anti-inflammatory activity of *C. sativa* burr (CSB) aqueous extract. The spectrophotometric study revealed high total phenolic content (TPC) values with significant antioxidant and anti-radical properties. Using UPLC-MS/MS techniques, the phytochemical investigation identified 56 metabolites, confirming the presence of phenolic compounds in CSBs. In addition, CSBs significantly downregulated pro-inflammatory mediators in LPS-stimulated RAW 264.7 macrophage cells without significant cell toxicity. Lastly, in silico studies pinpointed three kinases from RAW 264.7 cells as binding partners with ellagic acid, the predominant compound found in our extract. These findings strongly advocate for the recycling and valorization of *C. sativa* by-products, challenging their conventional classification as mere “waste”.

## 1. Introduction

*Castanea sativa* (Mill.), commonly known as the sweet chestnut, belongs to the family Fagaceae and is a key fruit crop in Southern Europe, bearing notable economic significance. Worldwide, its production is predominantly concentrated in the two macro-regions of Asia and Europe [1,2]. Italy stands as the leading chestnut producer in Europe, with five regions specializing in cultivation [3]. The *C. sativa* specimens investigated in this study originate from chestnut groves in Monte Amiata (Tuscany) and bear the “Protected Geographical Indication (PGI) Castagna del Monte Amiata” certification. Harvesting typically occurs from September to November [4], with most of the fruit earmarked for industrial processing, while the remainder is designated for fresh consumption. At this stage, a considerable quantity of protective spiny burrs surrounding the chestnuts is discarded as waste or burned to prevent pest infestation, which could harm future crops [5].

With an increasing focus on sustainable and circular economies, there is a rising interest in recovering bioactive molecules from waste and by-products within the agrifood and forestry supply chain. *C. sativa* main fruit or by-products’ extracts are a valuable source of bioactive secondary metabolites with outstanding antioxidant, anti-inflammatory, and anti-microbial properties [1,6,7,8,9,10,11]. These characteristics have led to the proposition of the application of *C. sativa* in the health and cosmetic industries, as demonstrated by several studies [1,12,13,14,15,16]. Currently, plant metabolites contribute significantly to the pharmaceutical industry’s revenue. Given the anticipated expansion of the global biomedical market to reach $232,280 million by 2028, the economic significance of employing plant materials for bioactive compounds has become considerable [17,18]. As our understanding of the immune system deepens, the pivotal role of inflammation in health outcomes and disease progression becomes increasingly evident. Inflammatory states impact not only the occurrence and prognosis of cancer but also influence gut dysbiosis and various other aspects of health, underscoring their wide-ranging effects [19,20,21]. Inflammation, particularly chronic inflammation, contributes to the development and progression of various diseases throughout life. While serving as an evolutionary defense mechanism against pathogens and tissue damage, persistent low-grade inflammation, or systemic chronic inflammation (SCI), is implicated in numerous age-related conditions, including cardiovascular disease, neurodegenerative disorders, cancer, diabetes, and autoimmune conditions, influenced by factors such as lifestyle choices, environmental exposures, chronic infections, and developmental origins [22]. The same lifestyle factors significantly influence inflammatory skin diseases such as psoriasis, atopic dermatitis, and contact dermatitis. Dietary components like gluten, along with lifestyle habits such as smoking and alcohol consumption, sleep patterns, and obesity, all play critical roles in exacerbating these conditions. For instance, research suggests that gluten consumption can worsen psoriasis and atopic dermatitis, while smoking is associated with increased severity and risk of these conditions. [23]. Therefore, there is an increasing need to explore novel anti-inflammatory agents, and natural sources, especially abundant waste materials, can offer valuable avenues for research and development in this regard. Chestnut by-products present an excellent opportunity to meet this demand while addressing the issue of waste reduction. Scientific research has revealed that chestnut by-products contain functional substances with antioxidant and anti-inflammatory properties [13,14,15], making them valuable and readily available raw materials for the pharmaceutical, nutraceutical, and cosmetic sectors. Among the compounds of interest, polyphenols have gained growing commercial value in the nutrition, cosmetic, and pharmaceutical industries. The global polyphenol market has experienced rapid growth in recent years, with an expected compound annual growth rate (CAGR) of 7.4% from 2023 to 2030 [24]. The potential benefits of utilizing crop waste as a natural source of polyphenols have been underscored by numerous reports [1,25,26,27], replacing expensive chemically synthesized antioxidants, anti-inflammatory, and artificial dye compounds.

The purpose of this study was to emphasize the potential for recovering added-value products from PGI *C. sativa* Monte Amiata spiny burrs, which can act as an innovative, cost-effective, and readily available raw material for applications in the health sector. An aqueous extract obtained from the spiny burrs of *C. sativa*, utilizing a total green ultrasound-assisted extraction method, was employed to assess the in vitro anti-inflammatory properties of chestnut spiny burrs. In silico studies were conducted to identify the anti-inflammatory target complement of RAW 264.7 cells, and docking simulations yielded insights into potential compounds within the aqueous extract from chestnut spiny burrs that interacted with the target. The ultimate objective was to extract essential secondary metabolites from these valuable by-products, rendering them a viable natural source for nutraceutical or cosmeceutical applications. The identified potential of *C. sativa* spiny burr extracts includes developing them into topical treatments for inflammatory skin conditions, natural supplements for managing chronic inflammatory diseases, and incorporating them into cosmetic products targeting skin inflammation and aging.

## 2. Materials and Methods

### 2.1. Materials

RPMI medium, Dulbecco’s Modified Eagle’s Medium (DMEM), trypsin-EDTA, and all the reagents used for cell culture were acquired from Merck (Darmstadt, Germany). Mouse immortalized fibroblasts (NIH3T3) and RAW 264.7 cells were from ATCC (Manassas, VA, USA). An Ames test kit was supplied from Xenometrix (Allschwil, Switzerland). Trolox was purchased from Sigma-Aldrich (St. Louis, MO, USA).

### 2.2. Preparation of C. sativa Burr (CSB) Extract 

The spiny burrs from *Castanea sativa* Mill., certified as PGI (Protected Geographical Indication) Castagna del Monte Amiata (Reg. CEE n. 2081/92), were sourced from Tuscany, a significant region for chestnut production in Italy. After being thoroughly cleaned, the material was dried at room temperature (RT) until it reached a constant weight. The pulverized spiny burrs were then powdered and kept in dark, sealed plastic bags at −80 °C until extraction. Extraction was performed in an ultrasonic bath using 20 kHz frequency ultrasounds on 330 g of pulverized chestnut spiny burrs, dispersed in 1 L of water, for three hours at room temperature. The obtained aqueous extract, designated as CSB, was freeze-dried in a lyophilizer (Lyovapor L-200, Bhuchi, India) and stored at −32 °C for further studies.

### 2.3. Total Phenolic Content (TPC)

The TPC was quantified using the Folin–Ciocalteu (FC) assay [28] with modifications. A calibration curve was generated using gallic acid (GA) solutions in the concentration range of 20–120 μg/mL. CSB samples were prepared by diluting the stock solution (1 mg/mL) in milli-Q water. Standard and sample tubes were then mixed with 1 mL of 1 N FC reagent in milli-Q water. After 3 min, 1 mL of saturated Na_2_CO_3_ and 7 mL of milli-Q water were added. All tubes were incubated for 90 min at room temperature, shielded from light, before measuring absorbance at 725 nm. Simultaneously, a solution containing all reagents with the extract solvent alone were prepared as blank. TPC was expressed as milligrams of GA equivalent (GAE) per gram of dry extract.

### 2.4. Total Flavonoid Content (TFC)

The TFC was determined using the aluminum chloride (AlCl_3_) method [29]. A calibration curve was generated using quercetin (Q) solutions in the concentration range of 20–200 μg/mL. CSB stock solution (1 mg/mL) was diluted in milli-Q water. Next, 500 μL of standard/sample was mixed to 100 μL of 10% AlCl_3_ in 1 M of potassium acetate and 3.3 mL of ethanol, in triplicate. Following 30 min of incubation, the absorbance was measured at 430 nm using an EnVision system (PerkinElmer). The results were expressed as mg of Q equivalent (QE) per gram of extract.

### 2.5. Determination of Reducing Power

The total reducing power (TRP) of CSB extracts was assessed using the potassium ferricyanide reducing power assay, following a modified version of the method described by [30]. A calibration curve was generated using ascorbic acid (AA) solutions in the concentration range of 20–140 μg/mL. CSB stock solution (1 mg/mL) was diluted in milli-Q water, in the concentration range of 25–200 µg/mL. A blank was created with water. 

The samples, standards, and blank were treated with 1 mL of 0.2 M phosphate buffer (K_2_HPO_4_:KH_2_PO_4_) at pH 6.6 and 1 mL of 1% potassium ferricyanide (K_3_[Fe(CN)_6_]), followed by incubation at 50 °C for 20 min. Subsequently, 1 mL of 10% (*w*/*v*) trichloroacetic acid was added to each solution, allowing an additional incubation at room temperature for 10 min. After this step, 2.5 mL of milli-Q water and 0.5 mL of 0.1% (*w*/*v*) ferric chloride (FeCl_3_) solution were added to 2.5 mL of the mixture before measuring the absorbance at 700 nm. The antioxidant power was quantified as mg AA equivalent (AAE) per gram of dry extract.

### 2.6. ABTS Free-Radical Scavenging Activity 

Trolox equivalent antioxidant capacity (TEAC) assay is based on the conversion of oxidized ABTS radicals to ABTS by molecules able to neutralize the radical [31]. The assay was performed using the OxiSelect™ TEAC Assay Kit (Cell Biolabs Inc., San Diego, CA, USA) according to the manufacturer’s instructions. Briefly, 25 µL of different concentrations of standard/sample (both in the concentration range of 2–75 µg/mL) were added to 150 µL of freshly prepared ABTS reagent diluted 1:50 in the appropriate diluent in a 96-well plate. Following 5 min incubation on an orbital shaker, the absorbance was measured at 405 nm. Results were expressed as IC50 (µg/mL) (i.e., Inhibitory Concentration causing a 50% decrease in the absorbance).

### 2.7. DPPH Free-Radical Scavenging Activity

DPPH free-radical scavenging activity was estimated by dosing the free DPPH (2,2-diphenyl-1-picrylhydrazyl) radical according to the method of Yen and Chen [32], with some modifications. Briefly, 100 µM of DPPH was added to each standard/sample dilution (both in the concentration range of 5–100 µg/mL), and the solutions were incubated 30′ in the dark, at 37 °C. The reaction was monitored at 517 nm to determine the percentage of discoloration. Τrolox (T) was used to set the standard curve. The capability to scavenge the DPPH radical was reported as IC50 (µg/mL) (i.e., Inhibitory Concentration causing a 50% decrease in the absorbance).

### 2.8. UPLC-MS/MS

The phytochemical composition of CSB was assessed through UPLC-MS/MS analysis using an Ultimate 3000 UPLC system, operated with Thermo Xcalibur software version 4.3.73.11 (Thermo Fisher Scientific, Waltham, MA, USA). The dry CSB extract was initially reconstituted in water and underwent protein precipitation, centrifugation, and desiccation of the resulting supernatant. It was then reconstituted to its original suspension volume (250 μL) before injection into the UPLC-Q-Exactive Plus system for analysis, as detailed in a previous study [33]. 

### 2.9. In Vitro Cytotoxicity and Anti-Inflammatory Activity

The ISO 10993-5:2009 [34] document (Biological evaluation of medical devices—Part 5: Tests for in vitro cytotoxicity). suggests several cell lines from the American Type Collection as models to evaluate the cytotoxicity of new materials or compounds. Among these, NIH3T3 mouse fibroblasts were selected to test the in vitro cytotoxicity of the CSB extract.

#### 2.9.1. Cell Cultures

NIH3T3 and RAW 264.7 macrophage cells were purchased from ATCC (ATCC, Manassas, VA, United States) and cultured in DMEM supplemented with 10% *v*/*v* Fetal Bovine Serum, 100 mg/mL of penicillin, and 100 mg/mL of streptomycin. The cultures were maintained at 37 °C in a humidified atmosphere with 5% CO_2_. Comparative analyses were conducted using cell populations at the same generation.

#### 2.9.2. NIH3T3 Cytotoxicity

When the NIH3T3 cells reached confluence, they were washed with 0.1 M of PBS (pH = 7.4), detached using a trypsin-EDTA solution, centrifuged at 1200 RCF for 5 min, and then re-suspended in complete medium at a density of 1.5 × 10^4^ cells/mL. The cells were then seeded into each well of 24-well plates and incubated at 37 °C in a 5% CO_2_ atmosphere. After 24 h of culture, the test compounds, appropriately diluted in complete medium, were added to each well. The following concentrations of CSB extract were tested: 0.025, 0.050, 0.10, and 10.0 mg/mL for 24, 48, and 72 h. The experiments were repeated three times, with all samples set up in six replicates. Complete medium served as the negative control. After 24, 48, and 72 h of incubation, cell viability was assessed using the neutral red uptake (NRU) assay as previously described [35].

#### 2.9.3. Cell Viability

RAW 264.7 cells were seeded at a density of 1×10^4^ cells per well in 96-well plates and cultured until they reached 80–85% confluence. The cells were then treated with varying concentrations (6, 12, 25, 50, and 100 µg/mL) of CSB, prepared in dimethyl sulfoxide (DMSO) (Sigma-Aldrich), and diluted in medium, ensuring that the final DMSO concentration remained below 0.1% *v*/*v* throughout the experiment. Controls were treated with 0.1% *v*/*v* DMSO, equivalent to the highest CSB concentration used. After 24 h of treatment, the cells were washed with sterile PBS, and 3-[4,5-dimethylthiazol-2-yl]-2,5-diphenyl tetrazolium bromide (MTT) was added to a final concentration of 1 mg/mL. Following a 2 h incubation, the cells were lysed with 150 µL of DMSO. Absorbance was measured at 550 nm using an EnVision system (PerkinElmer), and the percentage of cell viability was calculated relative to the vehicle control. 

#### 2.9.4. Cell Stimulation

Cells were treated with CSB prior to stimulation with Lipopolysaccharide (LPS) (obtained from Escherichia coli O111:B4, Sigma-Aldrich, St. Louis, MO, USA). Dexamethasone (DEX), a standard anti-inflammatory agent, was used as a positive control at a concentration of 5 µg/mL (Sigma-Aldrich). 

#### 2.9.5. Quantification of Intracellular ROS Generation

The generation of reactive oxygen species (ROS) in RAW 264.7 cells was determined in 96-well plates using 2′,7′-dichlorodihydrofluorescein diacetate (DCFH_2_-DA). This compound is deacetylated inside the cell and oxidized to the fluorescent 2′,7′-dichlorofluorescein (DCF) [36]. Cells were pre-treated with various concentrations of CSB (25, 50, and 100 µg/mL) before being stimulated with LPS (200 ng/mL) for 5 h. After this, DCFH_2_-DA (10 µM) dissolved in Hank’s Balanced Salt Solution was added to the cells and incubated for 10 min at 37 °C. The fluorescence was then measured using an EnVision system (PerkinElmer) with an excitation λ of 485 nm and an emission λ of 535 nm. To determine the number of cells in each well, a Crystal Violet assay was performed [37]. Briefly, after removing the medium, the cells were washed and stained with 0.1% crystal violet at RT for 20 min under stirring. Next, cells were washed and incubated with 200 µL of pure ethanol for another 20 min at RT under stirring. Optical density was measured at 570 nm. The results were normalized to the relative cell count for each well and expressed as the relative ROS production percentage (RFI) compared to the LPS group.

#### 2.9.6. Determination of NO Production

The production of nitric oxide (NO) in the supernatant of RAW 264.7 cells was determined in 6-well plates (1 × 10^6^ cells/well) cultured until sub-confluence (80–85%). After treatment with CSB at different concentrations (25, 50, and 100 μg/mL) for 4 h, the cells were stimulated with LPS (200 ng/mL) for 24 h. Following stimulation, 100 µL of conditioned medium from each well was transferred to a new 96-well plate and mixed with an equal volume of Griess reagent composed of 1% sulfanilamide and 0.1% N-(1-naphthyl) ethylenediamine dihydrochloride in 5% phosphoric acid. After incubation at RT for 10 min, the absorbance was measured at 540 nm using an EnVision system (PerkinElmer, Waltham, MA, USA). The nitrite concentration was assessed by a sodium nitrite standard curve.

#### 2.9.7. Protein Extraction

Whole-cell lysates were prepared using RIPA buffer supplemented with phosphate and protease inhibitors. The cell lysates were then sonicated for 15 min in an ice bath to facilitate disruption. Protein concentration was determined using a bicinchoninic acid protein (BCA) assay. For nuclear fractionation, the NE-PER™ Cytoplasmic and Nuclear Protein Extraction Kit (Thermo Fisher Scientific, Rockford, IL, USA) was utilized following the manufacturer’s instructions.

#### 2.9.8. Western Blotting 

A quantity of 20 µg of protein was resolved by 8% SDS-PAGE and transferred onto a nitrocellulose membrane. The membrane was blocked in PBS containing 10% *w*/*v* nonfat dry milk at room temperature with gentle shaking for 2 h. It was then incubated overnight at 4 °C with the following primary antibodies: anti-iNOS (rabbit polyclonal IgG, 1:10,000, Sigma-Aldrich), anti-NF-κB p65 (mouse monoclonal antibody, clone 1G10.2, 1:500, Sigma-Aldrich), and anti-GAPDH (glyceraldehyde-3-phosphate dehydrogenase) horseradish peroxidase (HRP)-conjugated (1:50,000). The membrane was washed three times and then incubated for 1 h at room temperature with HRP-conjugated secondary antibodies: anti-rabbit (1:80,000, Sigma-Aldrich) and anti-mouse (1:50,000, Sigma-Aldrich). After three more washes, immunoreactive bands were detected using Enhanced Chemiluminescence (Luminata Crescendo, Merck Millipore, Burlington, MA, USA), and images were acquired using the LAS4000 system (GE Healthcare, Chicago, IL, USA). The optical densities of the immunoreactive bands were analyzed using ImageQuant TL software (GE Healthcare, Chicago, IL, USA, V 7.0), with GAPDH serving as the loading control. 

#### 2.9.9. Immunofluorescence Study

RAW 264.7 cells were cultured on glass coverslips for 24 h. Subsequently, the cells were pre-treated with CSB at a concentration of 100 μg/mL for 4 h, followed by stimulation with LPS for 1 h. The cells were then fixed with 4% paraformaldehyde dissolved in PBS for 15 min. After three washes, the cells were permeabilized using 0.5% Triton X-100 in PBS for 5 min. Following permeabilization, the cells were incubated with a 5% solution of Normal Goat Serum (NGS) in PBS for 20 min, and subsequently with a 1:200 dilution of anti-NF-κB p65 (clone 1G10.2) mouse monoclonal antibody (Sigma-Aldrich) at 4 °C overnight. After three washes with PBS for 10 min each, the cells were incubated with a 1:100 dilution of Alexa 594-conjugated goat anti-Mouse IgG (Life Technologies, Carlsbad, CA, USA) for 1 h in the dark at room temperature. Following three washes with PBS and one wash with distilled water, the cells were mounted with a fluoroshield mounting medium containing 4′,6-Diamidino-2-Phenylindole (DAPI). Fluorescence microscopy (Zeiss AxioLabA1, Oberkochen, Germany) was used to capture images.

### 2.10. Mutagenicity Assay: Ames Test

The TA100 and TA98 strains of *Salmonella typhimurium* were utilized for the mutagenicity assay, both with and without metabolic activation (using the S9 liver fraction). These strains were chosen due to their sensitivity and capability to detect a significant number of known bacterial mutagens, making them a standard in the pharmaceutical industry [38]. The specific positive controls employed were 2-Nitrofluorene (2-NF) at 2 µg/mL and 4-Nitroquinoline N-oxide (4-NQO) at 0.1 µg/mL for tests without S9, and 2-aminoanthracene (2-AA) at 5 µg/mL for tests with S9. The S9 concentration in the culture was 4.5%.

Approximately 10^7^ bacteria were exposed to six different concentrations (0.025; 0.050; 0.10; 0.50; 1.0; and 10.0 mg/mL) of the CSB extract, as well as to positive and negative controls, for 90 min in a medium containing enough histidine to support roughly two cell divisions. After 90 min, the exposure cultures were diluted in a pH indicator medium without histidine and distributed into 48 wells of a 384-well plate. Within two days, cells that had reverted to His grew into colonies. Bacterial metabolism lowered the medium’s pH, altering the color of the wells, which could be visually observed. The number of wells containing revertant colonies was counted for each dose and compared to a zero-dose control. Each dose was tested in six replicates.

The material was deemed mutagenic if the number of histidine revertant colonies was at least twice that of the spontaneous revertant colonies. 

The specific positive controls used were 2-Nitrofluorene (2-NF) at 2 µg/mL and 4-Nitroquinoline N-oxide (4-NQO) at 0.1 µg/mL for tests without S9, and 2-aminoanthracene (2-AA) at 5 µg/mL for tests with S9. The final concentration of S9 in the culture was 4.5%.

### 2.11. Statistical Analysis

Experiments were performed in triplicate. Statistical analyses were performed with GraphPad Prism 9.0 software (GraphPad Software, San Diego, CA, USA). Data are presented as mean ± SD and were compared using the unpaired *t*-test or the one-way ANOVA with an appropriate post hoc test. A *p* value of 0.05 or less was considered significant.

### 2.12. In Silico Studies

#### Structural Optimization and Resources

The targets used in this study were obtained from DrugBank [39,40] using the “target section” with the keyword “inflammatory”, and analyzing the RAW 264.7 transcriptome through the Harmonizome 3.0 database [41], we selected the RAW 264.7 receptor complement. 3D structures were obtained following multiple sequence alignment (MSA) with BLASTp v.2.15.0 against the Protein Data Bank (PDB) database [40] using all other parameters as default [42], while the primary structures of targets were downloaded from the UniProt database [43]. All targets considered in this study are reported in Table S1. 

To optimize the docking simulations, the systems were improved as reported in previous work [44].

To improve the robustness of our in silico study, we performed docking simulations based on in vitro evidence. We considered only the targets where their experimental 3D structures in complex with an active compound were available (considering different binding regions, such as allosteric pockets), and we generated a box able to involve the binding regions. The docking simulation was executed set at exhaustiveness of 32, and all other parameters were set as default.

3D structures of compounds were obtained from the PubChem database [45] (detailed information provided in Table S2) and converted in pdbqt format, as proposed in previous works [46,47]. The PLIP tool [48] analyzed the interaction network, while potential key residues of targets were explored by using ClustalW v.2.1 [49] following MSA.

## 3. Results

### 3.1. Chemical Composition and Antioxidant Capacity of CSB

The extract of *C. sativa* burrs (CSBs) under investigation, produced by ultrasound-assisted extraction using water as a solvent, was first characterized for its TPC and TFC using appropriate colorimetric dosages to evaluate the extraction process’s efficiency. The antioxidant capacity of the extract was then evaluated by estimating the reducing power using the potassium ferricyanide method and the radical scavenging activity using the ABTS and DPPH assays. Table 1 shows the calculated TPC (mg GAE/g dry extract), TFC (mg QE/g dry extract), reducing power (mg AAE/g dry extract), and radical scavenging activity (IC50 µg/mL) of CSB. 

**Figure 1:**
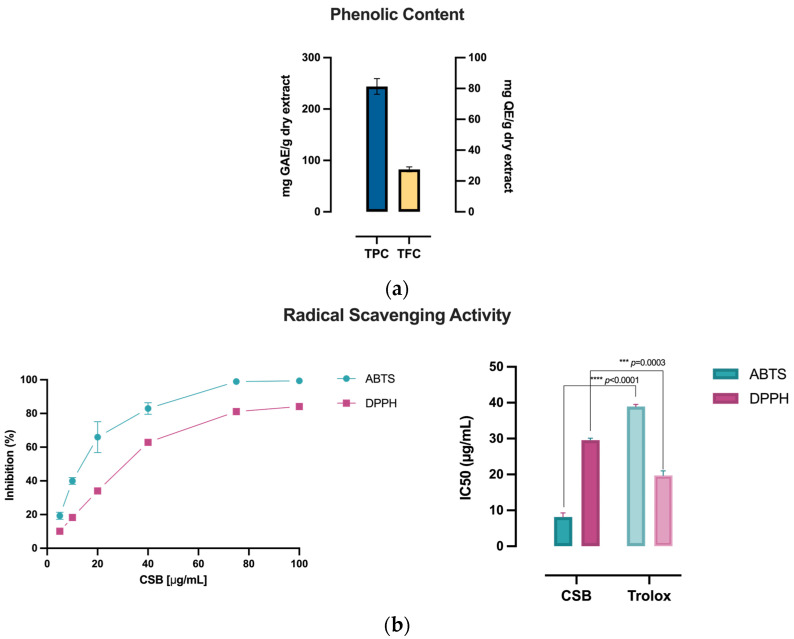
(**a**) Phenolic composition of CSB measured as TPC (mg GAE/g dry extract) and TFC (mg QE/g dry extract); (**b**) % Radical Scavenging Activity of CSB on ABTS and DPPH radicals with relative IC50 (µg/mL). All experiments were performed in triplicate. Data are presented as mean ± SD. Unpaired *t*-test was used to assess statistically significant differences, **** *p* < 0.0001, *** *p* = 0.0003, df = 2.

CSB exhibited an exceptionally elevated TPC of 243.98 ± 17.77 mg GAE/g, with a calculated TFC of 27.54 ± 0.60 mg QE/g (Table 1), as visually depicted in Figure 1a. Accordingly, CSB showcased notable reducing power, determined by the potassium ferricyanide method (272.12 ± 4.64 mg AAE/g of dry extract, Table 1), correlating with an elevated radical scavenging activity. Figure 1b illustrates a more pronounced impact on the ABTS radical compared to DPPH, notably revealing that CSB demonstrated an IC50 (µg/mL) (i.e., Inhibitory Concentration causing a 50% decrease in the absorbance) significantly lower (*p* < 0.0001) than Trolox, a water-soluble analog of vitamin E, employed as a standard compound. 

CSB was subsequently subjected to UPLC-MS/MS analysis. A total of 56 metabolites were identified using Compound Discoverer 3.3 software integrated with the ChemSpider database and the mzCloud for data processing and compared with the literature data. The results are listed in Table 2. 

### 3.2. NIH3T3 Viability and Proliferation

**Figure 2:**
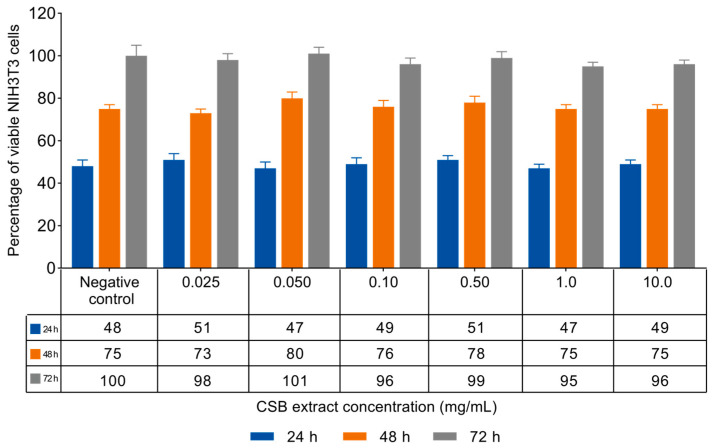
Percentage of viable NIH3T3 cells as a function of incubation time (24, 48, and 72 h) and CSB extract concentration, as determined by the neutral red uptake. Data are mean ± SD of three experiments run in six replicates. No value is statistically different versus negative control (complete medium), *p* < 0.05. *p*-values were calculated by *t*-test.

In vitro acute toxicity was evaluated by the NRU assay. Non-confluent adherent mouse fibroblasts (NIH3T3) were incubated with increasing concentrations of CSB extract ranging from 0.025 to 10 mg/mL. Cells were analyzed after 24, 48, and 72 h of incubation with the extract, and the results in terms of cell viability (%) as a function of both samples’ concentration and incubation time are reported in Figure 2.

The data reported in Figure 2 show that CSB extract was unable to influence the viability and proliferation of mouse fibroblasts at any of the tested concentrations and incubation times. ISO standard 10993-5:2009 [34] states that a material can be considered non-cytotoxic if it allows cell viability greater than 70% after exposure for 24 h. In this regard, CSB extract resulted as highly cytocompatible both in terms of viability and cell proliferation. 

### 3.3. CSB Inhibits LPS-Induced ROS Generation

**Figure 3:**
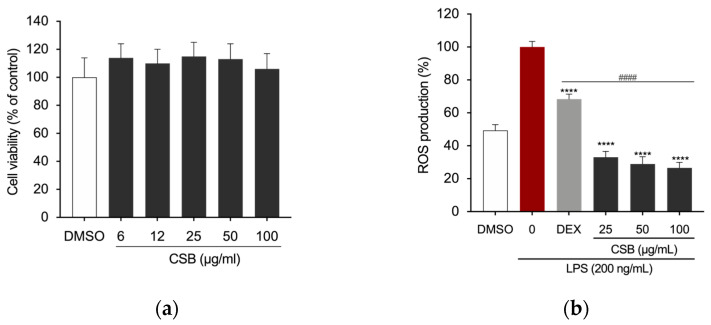
(**a**) Viability of RAW 264.7 cells after 24 h of treatment with CSB; (**b**) Intracellular ROS levels quantified after pre-treatment with various concentrations of CSB followed by LPS stimulation (200 ng/mL) for 5 h. Data are presented as bar graphs showing ROS level measured as relative fluorescence intensity normalized to cell count using the Crystal Violet assay. Statistically significant differences are denoted by **** *p* < 0.0001 (vs. LPS) or ^####^ *p* < 0.0001 (vs. DEX as positive control). All experiments were performed in triplicate. Data are expressed as a percentage of control and presented as mean ± SD. *p*-values were calculated by one-way ANOVA with Tukey’s post hoc test.

Cell viability of RAW 264.7 cells upon treatment with CSB was assessed by the MTT assay. Results are reported in Figure 3a as a percentage of cell viability compared to control, obtained by treating the cells with DMSO, as a vehicle, at the concentration corresponding to the highest dose used; the final concentration never exceeded 0.1% (*v*/*v*) in both treated and untreated cells and did not adversely affect the parameters analyzed. Results showed that no concentration of CSB used had an adverse effect on the cell viability of RAW 264.7 cells (Figure 3a). We used 25, 50, and 100 µg/mL as the optimal concentrations in the subsequent experiments. A microplate-based DCFH-DA assay showed that LPS-induced RAW 264.7 cells exhibited the highest fluorescence intensity DCFH-DA staining, which indicates ROS production; however, CSB significantly inhibited the cellular ROS generation at any tested concentration (Figure 3b). 

### 3.4. CSB Reduced LPS-Induced Inflammation in RAW 264.7 Cells

**Figure 4:**
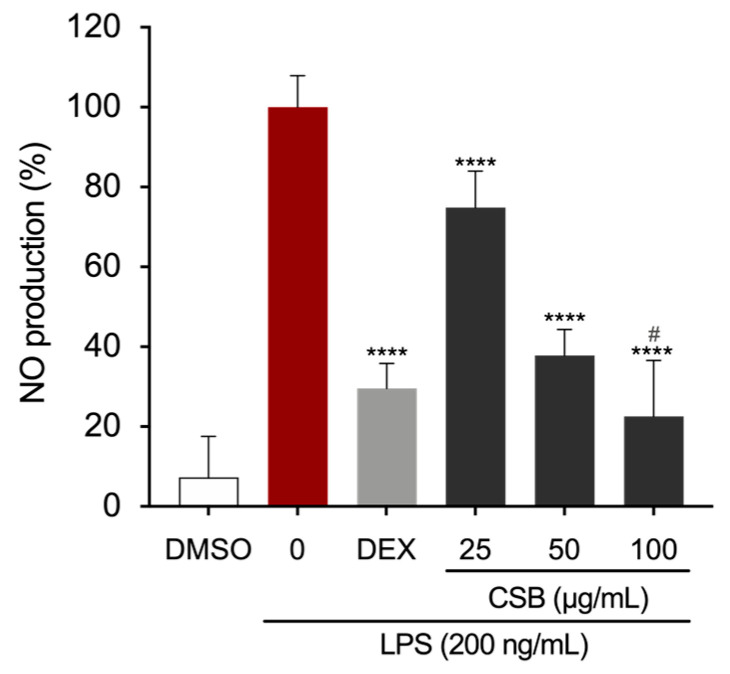
Effects of CSB on LPS-induced NO production in stimulated RAW264.7 cells. After pre-treating the cells with DEX or CSB for 4 h, the cells were stimulated with 200 ng/mL of LPS for 24 h. The culture supernatants were analyzed for NO production. Data show mean ± SD values of three independent experiments. **** *p* = 0.0001 (vs. LPS); ^#^ *p* = 0.0282 (vs. DEX as a positive control). *p*-values were calculated by one-way ANOVA with Tukey’s post hoc test.

Macrophages play a pivotal role in initiating the inflammatory cascade. To assess the anti-inflammatory properties of our chestnut burr extract, we treated LPS-stimulated RAW 264.7 murine macrophages with various concentrations of CSB and quantified the levels of key pro-inflammatory mediators. As expected, the NO production was strongly inhibited by DEX, which was used as a positive control at the concentration of 5 μg/mL. LPS-induced NO production was significantly decreased by the presence of CSB extract as well. The strongest effect was observed at 100 µg/mL, with significant reductions in NO levels also obtained at concentrations of 25 and 50 µg/mL (Figure 4). DMSO control demonstrated no effect on suppressing LPS-induced NO production in RAW264.7 cells. 

**Figure 5:**
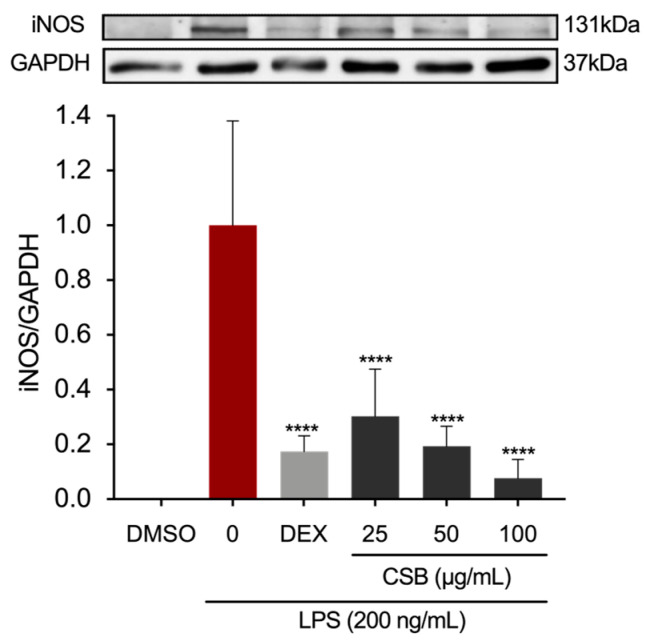
Effects of CSB on iNOS protein expression levels in stimulated RAW264.7 cells. After pre-treating the cells with DEX or CSB for 4 h, the cells were stimulated with 200 ng/mL of LPS for 24 h. The iNOS expression levels were determined by Western blotting. Data show mean ± SD values of three independent experiments. **** *p* < 0.0001 (vs. LPS). *p*-values were calculated by one-way ANOVA with Tukey’s post hoc test.

The protein expression of the precursor enzyme of NO, inducible nitric oxide synthase (iNOS), was assessed using Western blotting. Figure 5 shows a dose-dependent reduction of expression levels when compared to the LPS-treated group.

**Figure 6:**
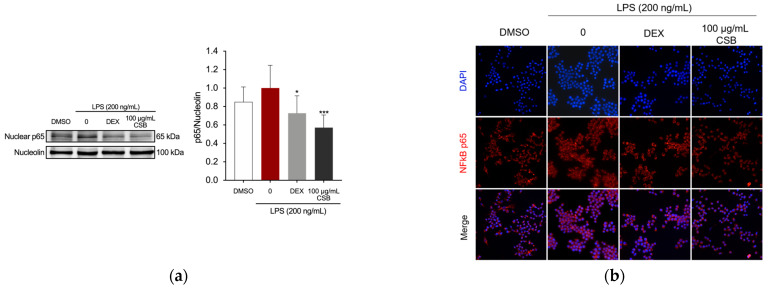
Effects of CSB on NF-κB activation in LPS-stimulated RAW264.7 cells. The cells were pre-treated with DEX or 100 μg/mL of CSB for 4 h, and then incubated with LPS (200 ng/mL) for 1 h. (**a**) NF-κB in LPS-stimulated RAW264.7 cells by Western blotting. Data are presented as mean ± SD of three independent experiments. * *p* = 0.0218, *** *p* = 0.0002 (vs. LPS). *p*-values were calculated by one-way ANOVA with Tukey’s post hoc test; (**b**) Localization of NF-κB visualized by a fluorescent microscope after staining for NF-κB (red). The nuclei of cells were stained with DAPI (blue). Micrographs were captured with 40× magnification.

We further explored whether CSB had the ability to interfere with the activation of Nuclear Factor kappa-light-chain-enhancer of activated B cells (NF-κB), which is involved in the regulation of inflammatory mediators in LPS-stimulated macrophages [50]. The analysis of NF-κB localization by immunostaining revealed that pre-treatment with the CSB extract significantly decreased the nuclear expression of NF-κB p65 in RAW 264.7 cells (Figure 6a). Fluorescence microscopy studies demonstrated that LPS stimulation induced the translocation of p65 into the nucleus; however, treatment with CSB extract at a concentration of 100 µg/mL retained NF-κB in the cytoplasm of the cells (Figure 6b).

### 3.5. Mutagenicity Assay: Ames Test

**Figure 7:**
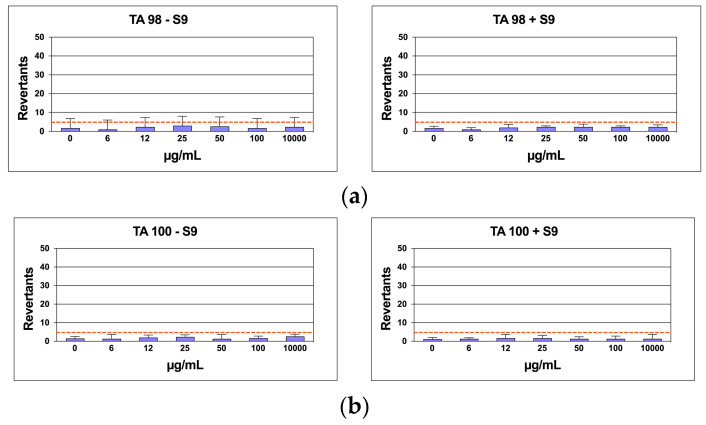
Number of revertants in TA98 (**a**) and TA100 (**b**) *S. typhimurium* strain exposed to different concentrations of CSB with S9 fraction and without S9 fraction. The results are reported as the mean of revertants ± SD, *n* = 6; *p* < 0.01.

In *Salmonella* mutagenicity assay, six different concentrations of the CSB extract were tested by Ames test on TA98, and TA100 strains with and without S9 metabolic activation. The results for the mutagenic effect of the samples, reported in Figure 7a,b, demonstrated that CSB extract at all the concentrations tested was not genotoxic towards both TA98 and TA100 with and without S9 fraction. In fact, also at the highest concentration (10 mg/mL), the number of revertants was lower and statistically different in comparison to positive control (*p* < 0.01). The background level, as well as positive control values, were in all cases within the normal limit found in our laboratory.

### 3.6. In Silico Results

#### Target/Compound Virtual Screening

**Figure 8:**
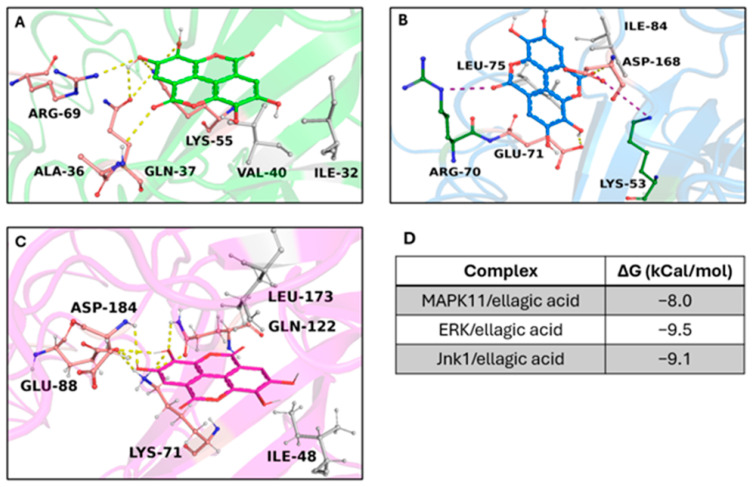
Overview of target/ellagic acid complexes. 3D structures of the crystal structure of (**A**) Jnk1 (PDB code: 3ELJ), (**B**) MAPK11 (PDB code: 8YGW), and (**C**) ERK1 (PDB code: 4QTB), are shown in the same complex with ellagic acid (shown in sticks) in green, blue, and magenta, respectively. The binding residues involved in hydrophobic interactions, hydrogen bonds, and salt bridges are represented as grey, pink, and depth green sticks, respectively. The hydrogen and salt bridge bonds are indicated as yellow and purple dotted lines. (**D**) The table reported the binding free energy (kCal/mol) of ellagic acid in complex with the targets.

To identify potential targets involved in interactions with our compounds, we conducted a ligand-based virtual screening against the entire RAW 264.7 cell anti-inflammatory target complement (41 targets indirectly/directly involved in the cell anti-inflammatory condition). A virtual screening was performed among targets and the compounds obtained from *C. sativa*. To standardize and reinforce in silico results, we applied two different strategies to select the best three complexes—(i) binding free energy (docking score) and (ii) evolution approach to identify consensus binding residues, as suggested in a previous work [44]. We selected the first three complexes with the highest binding free energy: the crystal structure of Jnk1 (PDB code: 3ELJ), the crystal structure of MAPK11 (PDB code: 8YGW), and the structure of human ERK1 (PDB code: 4QTB) showed the ability to strongly bind with ellagic acid (binding free energy from −9.5 Kcal/mol to −8 kcal/mol) triggering a large hydrophobic and polar interaction network. (Figure 8A–D). Multiple Sequence Analysis results revealed the ability of the compound to trigger strong polar interactions with target crucial residues, which are Lys-53 (ATP binding site), Glu-71 (inhibitor binding site) (residue number based on the crystal structure of MAPK11 with UniProtKB entry-Q15759-) (Figure S1), suggesting the potential inhibitor activity of the compound on the targets. 

## 4. Discussion

The demonstrated benefits of utilizing plant-derived metabolites, especially those obtained from waste biomasses, as a source of potential therapeutics are now widely acknowledged. The aim of this study was to extract bioactive compounds from the spiny burrs of Monte Amiata PGI *C. sativa* using a total-green ultrasound-assisted extraction method with water as the solvent. Traditionally, polyphenols and other antioxidant or potential therapeutic compounds are extracted using mixtures of methanol, ethanol, or acetone and water [51]. However, ultrasound-water extractions have shown promising efficiency in extracting phenolic compounds [52,53]. Ultrasound-assisted extraction, viewed through the lens of a circular bioeconomy, emerges as a green method with notable advantages over conventional techniques. It utilizes ultrasonic waves to induce cavitation bubbles in the extraction solvent, facilitating mass transfer and rupturing cell walls to release bioactive compounds [54]. This process operates at lower temperatures, preserving bioactivity and yielding shorter extraction times compared to traditional methods, which rely on heat and prolonged exposure to solvents [55]. Sustainability-wise, it eliminates the need for organic solvents, reducing environmental impact and making use of water as an eco-friendly solvent. With faster extraction rates and reduced operational expenses, ultrasound-assisted extraction proves to be an excellent choice for extracting bioactive substances from plant waste, offering a compelling blend of yield, sustainability, and practicality suitable for both laboratory and industrial applications. 

The effectiveness of the procedure was evaluated by measuring the TPC, TFC, and antioxidant capacity of the extract. Our findings indicated that CSBs demonstrated a notable abundance of phenolic compounds and displayed an overall antioxidant capacity, as assessed through various assays including TRP, ABTS, and DPPH. Notably, the radical scavenging activity of the ABTS radical exhibited by CSBs surpassed that of the standard Trolox, indicating a particularly robust antioxidant potential. These results suggest that the extraction method effectively retained a significant quantity of phenolic compounds in the CSB extract, contributing to its remarkable antioxidant properties. The superiority of CSB’s radical scavenging activity over the standard further emphasized its potential as a valuable source of natural antioxidants. The comprehensive assessment using multiple assays provides a robust understanding of the antioxidant capacity of the extract; however, reporting the phenolic content and antioxidant capacity of extracts in the literature is not standardized, making the comparative analysis challenging; this highlights the need for standardized normalization factors [51]. 

Since information is scarce in the literature regarding the aqueous extraction of *C. sativa* spiny burrs [1], and no in-depth investigations on the phytochemical composition of PGI *C. sativa* spiny burrs from Monte Amiata have been conducted, we analyzed CSB by UPLC-MS/MS, which confirmed the presence of high abundance of phenolic compounds, such as ellagic acid and other phenol glucoside derivatives, flavonoids and flavonol derivatives and their glycosides, as well as triterpenoids and plant hormones. Moreover, the analysis led to the identification of a wide range of secondary metabolites with numerous biological activities such as antioxidant, antiviral, and anti-inflammatory activities, among others, suggesting the potential for reusing these by-products as a natural source of bioactive phytochemicals [56]. 

Evaluating the toxicity and non-mutagenicity of plant extracts is crucial for guaranteeing the safety and effectiveness of biotechnological products derived from them. In our study, we conducted in vitro assessments on the cytotoxicity of CSB towards NIH3T3 mouse fibroblasts, confirming its non-cytotoxic nature. Additionally, we evaluated its mutagenicity toward *Salmonella* strains, establishing its non-mutagenic properties. 

Subsequently, we explored its potential as an anti-inflammatory agent in value-added products by testing it on LPS-challenged RAW 264.7 macrophage cells. Oxidative stress and inflammation are strictly related. In the inflammatory process, macrophages play pivotal roles such as antigen presentation, phagocytosis, and immunomodulation by generating a variety of cytokines and growth factors [50]. It is well established that iNOS plays an important role in inflammation. NO, a downstream signaling factor of iNOS, along with several other pro-inflammatory cytokines and chemokines, are key players in regulating immune and inflammatory responses, causing symptoms such as pain, fever, and edema [57]. NO, whose production is regulated by iNOS, is a potent reactive molecule involved in inflammatory responses observed in activated macrophages and at inflammatory sites [58]. Hence, therapeutic strategies aimed at targeting macrophages and the mechanisms in which they are involved could pave the way for the development of novel anti-inflammatory agents. Numerous experimental models have been established to facilitate the development of new anti-inflammatory drugs. Among these, the in vitro model utilizing LPS-stimulated RAW 264.7 cells is frequently highlighted in the literature for its effectiveness in exploring the anti-inflammatory properties of natural extracts and medications. This model has consistently demonstrated suitability and reliability as an in vitro approach for studying inflammation [59]. Results first showed that CSB did not affect the cell viability of macrophages at any tested concentration, demonstrating no cytotoxic effects. Moreover, pre-treatment with CSB notably decreased ROS and NO production in the supernatants of RAW 264.7 cells and LPS-induced increased levels of iNOS protein in a dose-dependent manner. NF-κB plays a crucial role in regulating the gene encoding pro-inflammatory cytokines and inducible enzymes, including iNOS [50]. When the NF-κB pathway is activated, it drives the production of downstream inflammatory-related factors. Given that CSB affects NF-κB downstream pro-inflammatory markers, we investigated whether the extract could inhibit this pathway by evaluating its capacity to suppress LPS-induced translocation of NF-κB into the nucleus of RAW 264.7 cells. Our findings indicated that treating the cells with CSB resulted in a significant reduction in the expression of the p65 subunit of NF-κB into the nucleus. This suggests that CSB may effectively block the signal transduction pathways mediated by NF-κB.

Our in silico investigations, directed at pinpointing potential targets in RAW 264.7 cells implicated in anti-inflammatory conditions, supported the existing literature. We retrieved the complete anti-inflammatory target complex from the “target section” within the DrugBank database. Subsequently, we conducted molecular modeling to acquire and refine the 3D structures of the pre-selected targets. To define the potential compound(s) derived from chestnut spiny burrs possessing anti-inflammatory proprieties and its biological target(s), we carried out a virtual screening of our compounds. Ellagic acid emerged as the compound with the highest binding free energy score among all compounds on the best three targets, forming strong polar interactions with the target’s critical residues [60,61]. Furthermore, ellagic acid shared both a similar binding region and pose with known experimental inhibitors of these kinases.

Ellagic acid has gained much attention for its potential therapeutic effects in treating human diseases. Its properties include antioxidant, anti-inflammatory, antimutagenic, and antiproliferative. Alone, or combined with other antioxidants, it has shown positive therapeutic effects [62]. Other phenolic compounds identified in CSB were gallic acid and derivatives, especially n-propyl gallate and ethyl gallate. Gallic acid has been acknowledged for its therapeutic properties, such as its ability to act as an antioxidant and anti-inflammatory agent [63]. According to Mard et al., gallic acid pre-treatment of a gastric mucosal lesion decreased inflammatory responses via inhibiting iNOS [64]. Moreover, another phenolic compound found in CSB, Protocatechuic aldehyde (PCA), was previously reported to suppress inflammatory effects. More specifically, it was found that PCA reduced the production of NO and the expression level of the iNOS gene induced by LPS in RAW 264.7 cells [1]. Overall, numerous studies have reported the ability of phenolic compounds to repress inflammation-related genes in various types of cells [65,66,67,68,69].

Flavonoids was the main subclass of phenolic compounds in CSB. Reports have highlighted the remarkable anti-inflammatory effects of flavonoids, which are achieved by modulating the expression of pro-inflammatory molecules such as iNOS and proinflammatory cytokines [65,66,70,71]. Research indicates that the consumption of flavonoids through diet is linked inversely to age-related diseases such as cardiovascular disease, neurodegeneration, and type 2 diabetes [70,71]. Moreover, Lim et al. reported studies demonstrating that uptake of flavonoids, including kaempferol, also lowered the elevated level of inflammatory cytokines and NF-κB activation in aged animal model [66]. Worth mentioning is the presence of betaine in CSB, also known as trimethylglycine, as it is a modified amino acid that is considered an important human nutrient [72]. This dietary supplement obtained from various foods has demonstrated anti-inflammatory potential [73,74]. Go et al. conducted a study on Sprague–Dawley (SD) rats to evaluate the in vivo anti-inflammatory effect of betaine on NF-κB. The results showed that betaine inhibited NF-κB and the expression of related genes expression of iNOS and attenuated oxidative stress-induced NF-κB in YPEN-1 cells. This suggested its potential to prevent NF-κB activation during aging and inflammation [74].

Although some compounds were already reported in the spiny burrs of *C. sativa* [13,14,25,56], to the best of our knowledge, this is the first report on the detailed composition of *C. sativa* spiny burrs from Monte Amiata. The high antioxidant potential of chestnut burr extracts is due to their phenolic contents, commonly extracted using hydroalcoholic solvents [51]. Nonetheless, we found high content of phenolic compounds in *C. sativa* spiny burrs extracted with an innovative water-ultrasound method, which has the advantages of being non-toxic, environmentally friendly, and safe. It is worth mentioning that other extracts from *C. sativa* by-products have demonstrated anti-inflammatory potential. An extract tested on BV-2 microglia cells showed cytoprotective activity and reduction of the transcriptional levels of cytokines, and NF-kB expression following LPS stimulation, imputable to the presence of flavonoids such as astragalin, isorhamnetin glucoside, and myricitrin [25]. Furthermore, GA and PCA emerged as the predominant phenolic compounds within a chestnut shell extract that demonstrated effective protection against inflammation, dehydration, and photoaging. This efficacy was evidenced through the assessment of protein expression related to water balance and collagen stability in human keratinocytes [1].

The discovery that an aqueous extract from the spiny burrs of *C. sativa*, rich in antioxidant compounds, can significantly suppress the main inflammatory players in macrophagic cells opens many possibilities for treating various inflammatory illnesses. CSB, abundant in polyphenols, efficiently inhibits ROS production in LPS-stimulated macrophages, offering potential protection against oxidative stress-induced cellular damage. This observation is consistent with the well-established antioxidant properties of polyphenolic compounds, including flavonoids, present in CSB. Their collective contribution underscores the extract’s substantial antioxidative capacity, thereby accentuating its promising utility in the development of advanced biotechnological formulations aimed at addressing conditions associated with oxidative stress.

## 5. Conclusions

Our research provides valuable information regarding the phytochemical composition, antioxidant capacity, and therapeutic properties of *C. sativa* spiny burrs with PGI status from Monte Amiata. The bioactive compounds extracted using water and ultrasounds demonstrate antioxidant activities and anti-inflammatory properties. This innovative method offers the advantages of scalability, precise control over extraction parameters, and lower maintenance costs. This promising outcome suggests that these by-products could be employed in bio-products as a natural source of beneficial compounds. The high phenolic content, extracted with a totally green method, is noteworthy due to its non-toxic, environmentally friendly, and safe nature. This perspective on waste valorization offers a solution to the economic and environmental challenges associated with the disposal of chestnut by-products, potentially generating income. Our findings strongly encourage the recycling and valorization of *C. sativa* by-products, which should no longer be considered as mere “waste”. The final objective is to highlight the potential for added-value product recovery from *C. sativa* Monte Amiata IGP spiny burrs, which can serve as an innovative, low-cost, and readily available raw material for application in the health sector.

## Figures and Tables

**Table 1:** TPC, TFC, and antioxidant capacity of CSB.

			Antioxidant Capacity		
	TPC (mg GAE/g)	TFC (mg QE/g)	TRP (mg AAE/g)	ABTS (IC50 µg/mL)	DPPH (IC50 µg/mL)
CSB	243.98 ± 17.77	27.54 ± 0.60	272.12 ± 4.64	8.16 ± 1.11	29.57 ± 0.57

Note: *TPC* Total Phenolic Content; *TFC* Total Flavonoid Content; *TRP* Total Reducing Power; *ABTS* 2,2′-azino-bis(3-ethylbenzothiazoline-6-sulfonic acid); *DPPH* 2,2-diphenyl-1-picrylhydrazyl; *GAE* Gallic Acid Equivalent; *QE* Quercetin Equivalent; *AAE* Ascorbic Acid Equivalent. Data are expressed as mean ± SD (*n* = 3).

**Table 2:** Matched metabolites in CSB US-water extract, along with their retention time, molecular formulae, observed and theoretical m/z, and error (ppm). For all matched compounds, the error was lower than 5 ppm.

No.	Name	Retention Time (min)	Formula	Calculated MW	Theoretical m/z	Reference Ion	Mass Error (ppm)	Peak Area (%)
1	Ellagic acid	15.388	C14H6O8	302.00617	300.9988	[M − H]^−1^	−0.33	51.7
2	Betaine	1.863	C5H11NO2	117.07923	118.0865	[M + H]^+1^	2.17	22.0
3	5,7-dihydroxy-3.8-dimethoxy-2-phenyl-4h-chromen-4-one	29.172	C17H14O6	314.07985	315.0871	[M + H]^+1^	2.59	15.8
4	Mollioside	25.285	C26H40O10	512.26256	513.2698	[M + H]^+1^	0.81	1.7
5	(±)-(2e)-abscisic acid	11.733	C15H20O4	264.13625	265.1435	[M + H]^+1^	0.34	1.6
6	3,8-di-o-methylellagic acid	21.233	C16H10O8	330.03838	331.0457	[M + H]^+1^	2.46	1.4
7	12-hydroxyjasmonic acid	18.885	C12H18O4	226.12108	227.1284	[M + H]^+1^	2.53	0.9
8	Epi-jasmonic acid	15.442	C12H18O3	210.12618	211.1335	[M + H]^+1^	2.77	0.7
9	Protocatechuic aldehyde	6.164	C7H6O3	138.03177	139.0391	[M + H]^+1^	0.56	0.4
10	Gibberellin A2 o-beta-d-glucoside	23.076	C25H36O11	512.22551	513.2328	[M + H]^+1^	−0.49	0.3
11	Sinapaldehyde	21.782	C11H12O4	208.07372	209.081	[M + H]^+1^	0.76	0.3
12	12-hydroxyjasmonic acid 12-o-beta-d-glucoside	19.078	C19H30O8	386.1931	387.2004	[M + H]^+1^	−2.5	0.3
13	5,7-dihydroxy-3′,4′,5′-trimethoxyflavanone	18.774	C18H18O7	346.10628	347.1136	[M + H]^+1^	2.97	0.2
14	(+)-Gibberellic acid	16.236	C19H22O6	346.14242	347.1497	[M + H]^+1^	2.25	0.2
15	N-propyl galiate	10.343	C10H12O5	212.06876	235.058	[M + Na]^+1^	1.35	0.2
16	Syringaldehyde	12.269	C9H10O4	182.05821	183.0655	[M + H]^+1^	1.68	0.2
17	Retusin (flavonol)	18.648	C19H18O7	358.10628	359.1136	[M + H]^+1^	2.87	0.2
18	Acaciin	11.453	C28H32O14	592.17833	593.1856	[M + H]^+1^	−1.48	0.2
19	Kaempferol	17.111	C15H10O6	286.04805	287.0553	[M + H]^+1^	1.1	0.2
20	Scopoletin	16.019	C10H8O4	192.04282	193.0501	[M + H]^+1^	2.91	0.2
21	Isorhamnetin 3-rhamnosyl-(1->2)-gentiobiosyl-(1->6)-glucoside	28.24	C40H52O26	948.27548	949.2828	[M + H]^+1^	0.84	0.1
22	5,7-methoxyflavanone	12.653	C17H16O4	284.10549	285.1128	[M + H]^+1^	2.21	0.1
23	4′.5.7-trimethoxyflavone	20.486	C18H16O5	312.10053	313.1079	[M + H]^+1^	2.44	0.1
24	Ethyl gallate	13.29	C9H10O5	198.05319	199.0605	[M + H]^+1^	1.87	0.1
25	2-(2,6-dimethoxyphenyl)-5,6-dimethoxy-4h-chromen-4-one (zapotin)	16.541	C19H18O6	342.11131	343.1186	[M + H]^+1^	2.85	0.1
26	Helichrysoside	20.13	C30H26O14	610.13403	633.1237	[M + Na]^+1^	2.91	0.1
27	1,4-dihydro-4-oxo-3-(2-pyrrolidinyl)-2-quinolinecarboxylic acid	17.834	C14H14N2O3	258.10066	259.1079	[M + H]^+1^	0.86	0.1
28	Afrormosin	16.195	C17H14O5	298.08489	299.0922	[M + H]^+1^	2.56	0.1
29	Gibberellin A17	6.043	C20H26O7	378.16813	377.1609	[M − H]^−1^	0.75	0.1
30	Quercetin	17.258	C15H10O7	302.04329	303.0506	[M + H]^+1^	2.11	0.1
31	1,3-bis-(5-carboxypentyl)-urea	11.099	C13H24N2O5	288.16887	289.1762	[M + H]^+1^	1.21	0.1
32	5-carboxyvanillic acid	13.026	C9H8O6	212.0318	211.0245	[M − H]^−1^	−1.37	tr.
33	3-hydroxyflavone	18.639	C15H10O3	238.06363	239.0709	[M + H]^+1^	2.68	tr.
34	Coniferaldehyde	19.593	C10H10O3	178.0632	179.0705	[M + H]^+1^	1.18	tr.
35	Gibberellin A1/A34	39.565	C19H24O6	348.15815	349.1654	[M + H]^+1^	2.47	tr.
36	Isorhamnetin 3-o-alpha-l-[6′-p-coumaroyl-beta-d-glucopyranosyl-(1->2)-rhamnopyranoside]	20.825	C37H38O18	770.20801	793.1978	[M + Na]^+1^	2.85	tr.
37	Gibberellin A53	38.444	C20H28O5	348.19405	349.2013	[M + H]^+1^	1.07	tr.
38	2′,5-digalloylhamamelofuranose	12.064	C20H20O14	484.08397	485.0913	[M + H]^+1^	−2.76	tr.
39	(+)-Catechin 7-o-beta-d-xyloside	18.011	C20H22O10	422.12248	445.1117	[M + Na]^+1^	2.81	tr.
40	Digallic acid	2.807	C14H10O9	322.03294	321.0257	[M − H]^−1^	1.43	tr.
41	(E)-ferulic acid	29.676	C10H10O4	194.05788	195.0652	[M + H]^+1^	−0.16	tr.
42	1,3-dibutyl-1,3-dimethylurea	13.625	C11H24N2O	200.18829	199.181	[M−H]^−1^	−2.86	tr.
43	Kaempferol-3-o-(6′-trans-p-coumaroyl-2′-glucosyl)rhamnoside	21.66	C36H36O17	740.19518	741.2025	[M + H]^+1^	−0.1	tr.
44	Tomentosin	15.583	C15H20O3	248.14121	249.1487	[M + H]^+1^	−0.15	tr.
45	Catechin gallate. (-)-	18.618	C22H18O10	442.08926	443.0965	[M + H]^+1^	−1.68	tr.
46	Coniferyl aldehyde	24.95	C10H10O3	178.0632	179.0705	[M + H]^+1^	1.18	tr.
47	Quercetin-3-o-(6′-trans-p-coumaroyl-2′-glucosyl)rhamnoside	18.932	C36H36O18	756.19158	779.1812	[M + Na]^+1^	1.87	tr.
48	Gibberellin A24	37.98	C20H26O5	346.17818	347.1855	[M + H]^+1^	0.45	tr.
49	Glucogallin	3.747	C13H16O10	332.07488	331.0676	[M − H]^−1^	1.61	tr.
50	5′-desgalloylstachyurin	11.878	C34H24O22	784.0757	783.0684	[M − H]^−1^	−0.29	tr.
51	Gibberellin A12	44.209	C20H28O4	332.19787	333.2052	[M + H]^+1^	−2.67	tr.
52	(+)-Gallocatechin	2.929	C15H14O7	306.07341	305.0661	[M − H]^−1^	−1.78	tr.
53	Isorhamnetin	23.872	C16H12O7	316.05908	315.0518	[M − H]^−1^	2.47	tr.
54	Myricetin-3-o-glucoside	8.465	C21H20O13	480.09172	479.0844	[M − H]^−1^	2.77	tr.
55	1,6-bis-o-galloyl-beta-d-glucose	8.974	C20H20O14	484.0862	483.0789	[M − H]^−1^	1.84	tr.
56	Castalagin/vescalagin	15.21	C41H26O26	934.06952	933.0623	[M − H]^−1^	−1.83	tr.
Tot								100

tr. = traces (Area < 0.1%).

## Data Availability

The original contributions presented in the study are included in the article/supplementary material, further inquiries can be directed to the corresponding author.

## Supplementary Materials

The following supporting information can be downloaded at: https://www.mdpi.com/article/10.3390/life14060763/s1, Figure S1: Sequence alignment results between human MAPK11, ERK1, and JNK1. Identical and positively conserved amino acid residues are marked with a star and colons, respectively. The red boxes highlight all the amino acids involved in hydrogen bonds and/or salt bridges with ellagic acid.; Table S1: List of targets considered in this study; Table S2: List of compounds considered in this study.
