# Supplementary material for: Repurposing Castanea sativa Spiny Burr By-Products Extract as a Potentially Effective Anti-Inflammatory Agent for Novel Future Biotechnological Applications
Source: Life (Basel). 2024 Jun 15;14(6):763. doi: 10.3390/life14060763 (PMC11205080; doi:10.3390/life14060763)
Supplement: Supplementary file 1 [file life-14-00763-s001.zip › Table S1.pdf]

Target Name	UniProt ID and entry Name	PDB Code
C-C motif chemokine 3	P10147 · CCL3_HUMAN	5COR
Mitogen-activated protein kinase 14	Q16539 · MK14_HUMAN	6ZWP
Triggering receptor expressed on myeloid cells 1	Q9NP99 · TREM1_HUMAN	1SMO
Arachidonate 5-lipoxygenase	P09917 · LOX5_HUMAN	7TTK
Mitogen-activated Protein Kinase 11	Q15759 · MK11_HUMAN	4QTB
Mitogen-activated protein kinase 3	P27361 · MK03_HUMAN	3ELJ
Mitogen-activated protein kinase 8	P45983 · MK08_HUMAN	1CJY
Cytosolic phospholipase A2	P47712 · PA24A_HUMAN	3HOF
Interleukin-1 alpha	P01583 · IL1A_HUMAN	5UC6
Annexin A1	P04083 · ANXA1_HUMAN	1AIN
Methyl dehydroabietate	Q9NR96 · TLR9_HUMAN	8AR3
Apigenin	P35354 · PGH2_HUMAN	5IKR
Interleukin-8	P10145 · IL8_HUMAN	6N2U
Interleukin-23 subunit alpha	Q9NPF7 · IL23A_HUMAN	5MJ3
cAMP-specific 3',5'-cyclic phosphodiesterase 4A	P27815 · PDE4A_HUMAN	2QYK
Receptor-interacting serine/threonine-protein kinase 1	Q13546 · RIPK1_HUMAN	4NEU
Interleukin-12 subunit beta	P29460 · IL12B_HUMAN	5MJ3
Serine/threonine-protein kinase TBK1	Q9UHD2 · TBK1_HUMAN	6RSU
Toll-like receptor 6	Q9Y2C9 · TLR6_HUMAN	4OM7
72 kDa type IV collagenase	P08253 · MMP2_HUMAN	1RTG
Prostaglandin G/H synthase 1	P23219 · PGH1_HUMAN	6Y3C
Eicosapentanoic acid C20:5	P35354 · PGH2_HUMAN	5F19
Docosahexaenoic acid C22:6	O60603 · TLR2_HUMAN	8AR0
Glyceraldehyde-3-phosphate dehydrogenase	P04406 · G3P_HUMAN	1U8F
High mobility group protein B1	P09429 · HMGB1_HUMAN	2YRQ
MAP kinase-activated protein kinase 2	P49137 · MAPK2_HUMAN	1KWP
Tyrosine-protein kinase Fgr	P09769 · FGR_HUMAN	7JT9
CCAAT/enhancer-binding protein beta	P17676 · CEBPB_HUMAN	7UPZ
Macrophage colony-stimulating factor 1 receptor	P07333 · CSF1R_HUMAN	6T2W
Tyrosine-protein kinase SYK	P43405 · KSYK_HUMAN	4FL2
Tyrosine-protein kinase Lyn	P07948 · LYN_HUMAN	6NMW
