# Supplementary material for: Repurposing Castanea sativa Spiny Burr By-Products Extract as a Potentially Effective Anti-Inflammatory Agent for Novel Future Biotechnological Applications
Source: Life (Basel). 2024 Jun 15;14(6):763. doi: 10.3390/life14060763 (PMC11205080; doi:10.3390/life14060763)
Supplement: Supplementary file 1 [file life-14-00763-s001.zip › Table S2.pdf]

Compound Name	Compound CID
Ellagic acid	5281855
Betaine	247
5.7-dihydroxy-3.8-dimethoxy-2-phenyl-4h-chromen-4-one	9972910
Mollioside	28287201
(±)-(2e)-abscisic acid	5375199
3.8-di-o-methylellagic acid	5488919
12-hydroxyjasmonic acid	5497122
Epi-jasmonic acid	7251183
Protocatechuic aldehyde	8768
Gibberellin A2 o-beta-d-glucoside	16019961
Sinapaldehyde	5280802
12-hydroxyjasmonic acid 12-o-beta-d-glucoside	11966210
5.7-dihydroxy-3'.4'.5'-trimethoxyflavanone	61988
(+)-Gibberellic acid	6466
N-propyl galiate	4947
Syringaldehyde	8655
Retusin	5352005
Acaciin	5317025
Kaempferol	5280863
Scopoletin	5280460
Isorhamnetin 3-rhamnosyl- (1->2) -gentiobiosyl- (1->6) -glucoside	44259397
5.7-methoxyflavanone	103143244
4'.5.7-trimethoxyflavone	79730
Ethyl gallate	13250
zapotin	629965
Helichrysoside	5317991
1.4-dihydro-4-oxo-3-(2-pyrrolidinyl)-2-quinolinecarboxylic acid	69114
Afrormosin	5281704
Gibberellin A17	5460657
Quercetin	5280343
1.3-bis-(5-carboxypentyl)-urea	246663
5-carboxyvanillic acid	16488
3-hydroxyflavone	11349
Coniferaldehyde	5280536
Gibberellin A1/A34	5280379
Isorhamnetin 3-o-alpha-l-[6''''-p-coumaroyl-beta-d-glucopyranosyl-(1->[illegible]	11018161
Gibberellin A53	440914
2'.5-digalloylhamamelofuranose	253775
(+)-Catechin 7-o-beta-d-xyloside	73533
Digallic acid	341
(E)-ferulic acid	445858
1.3-dibutyl-1.3-dimethylurea	546287
Kaempferol-3-o-(6'''-trans-p-coumaroyl-2''-glucosyl)rhamnoside	10032927
Tomentosin	155173
Catechin gallate. (-)-	65064
Quercetin-3-o-(6'''-trans-p-coumaroyl-2''-glucosyl)rhamnoside	10169367
Gibberellin A24	443454
Glucogallin	124375
5'-desgalloylstachyurin	10417809
Gibberellin A12	443450
(+)-Gallocatechin	65084

Isorhamnetin	5281654
Myricetin-3-o-glucoside	22841567
1.6-bis-o-galloyl-beta-d-glucose	440221
Vescalagin	168165
