# Supplementary figures and images for: Repurposing Castanea sativa Spiny Burr By-Products Extract as a Potentially Effective Anti-Inflammatory Agent for Novel Future Biotechnological Applications
Source: Life (Basel). 2024 Jun 15;14(6):763. doi: 10.3390/life14060763 (PMC11205080; doi:10.3390/life14060763)

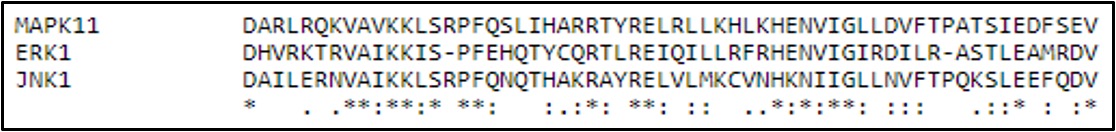

Supplement: Supplementary file 1 [file life-14-00763-s001.zip › Figure S1.jpg]
